# The Impact of a High-Altitude Expedition on the Physical Performance and Nutritional Indices of Health Status of Alpinists

**DOI:** 10.3390/jfmk10020143

**Published:** 2025-04-25

**Authors:** Ewa Karpęcka-Gałka, Marek Bawelski, Aleksandra Pięta, Paulina Mazur-Kurach, Paweł Pięta, Barbara Frączek

**Affiliations:** 1 Doctoral School of Physical Culture Sciences, University of Physical Culture, Jana Pawła II 78, 31–571 Cracow, Lesser Poland, Poland; 2 Department of Physiology and Biochemistry, Institute of Biomedical Sciences, University of Physical Culture, Jana Pawla II 78, 31-571 Cracow, Lesser Poland, Poland; 3 Department of Sports Medicine and Human Nutrition, Institute of Biomedical Sciences, University of Physical Culture, Jana Pawla II 78, 31-571 Cracow, Lesser Poland, Poland

**Keywords:** alpinism, body composition, climbing, health status, mountaineering, physical performance

## Abstract

**Objective:** The aim of the study was to determine the effect a mountain expedition (>3000 m) would have on the physical performance and nutritional indices of alpinists’ health status. **Methods:** The study included 17 men aged 30.29 ± 5.8 years participating in mountain expeditions to peaks of 5000–8000 m, lasting an average of 34 ± 6 days. The following were assessed: aerobic and anaerobic capacity, body composition and the values of selected biochemical and hematological indices of blood and urine before and after returning from the expeditions and a quantitative analysis of the alpinists’ diet. **Results:** There was a statistically significant decrease (*p* ≤ 0.05) in aerobic capacity, anaerobic capacity, subjects’ body mass, muscle mass and the lean body mass of the upper and lower extremities. There was a significant increase (*p* ≤ 0.05) in erythrocytes, hemoglobin, hematocrit, leukocytes, platelets, neutrophils, monocytes and a significant decrease (*p* ≤ 0.05) in total and high-density lipoprotein cholesterol, total bilirubin, albumin and total protein. A small percentage of the subjects met the requirements for iron (29.41%), folate (35.29%) and vitamin D (17.65%) supply with diet, as reflected in the blood test results. **Conclusions:** Despite the observed positive effect of three-week hypoxic exposure on the climbers’ health, the deterioration of aerobic and anaerobic capacity was shown, which, in addition to environmental conditions and systemic inflammation, may have been influenced by adverse changes in body composition. To improve the nutritional status of the body during the expedition and upon return, alpinists should consider including the necessary supplementation of deficient components.

## 1. Introduction

Alpinism is a discipline combining rock climbing, ice climbing, ski mountaineering and moving on glaciers [1]. A special variety of climbing is Himalayanism—that is, climbing practiced in the highest mountains in the world whose highest peaks exceed 7000 m—these are the mountain ranges of the Himalayas, Karakorum, Daxue Shan, Hindu Kush, Kunlun, Pamir and Tianshan [2]. Interest in mountaineering is growing every year, also among amateur climbers [3]. It is an endurance sport with elements of high-intensity strength training. Exercise typically involves short periods of high intensity and long periods of submaximal intensity that require anaerobic and aerobic energy pathways [4]. During climbing, oxygen consumption and heart rate increase, suggesting that it requires an optimal level of aerobic capacity [5,6]. In the high-altitude environment, mountaineers must contend with intense sunlight, heavy rain or snow and strong winds and temperature drops (an average of 0.6° for every 100 m of altitude) with large fluctuations throughout the day, as well as the risk of avalanches and falling rocks [1,7]. Staying in hypobaric hypoxia (>3000 m) causes a number of deleterious effects on alpine climbers, such as a decreased exercise capacity [8], impaired post-exercise recovery [9], sleep deprivation [10], cognitive impairment [11] and body mass loss [12]. Acclimatization to long-term hypoxia is regulated at the molecular level by hypoxia-inducible factor-1 (HIF-1), which is responsible for mobilizing hundreds of genes in response to oxygen limitation [13]. Table 1 shows the characteristics of the altitude ranges referred to in the article [14,15].

A number of studies have evaluated the aerobic and anaerobic capacity of Polish professional athletes [16,17,18,19,20], as well as the effect of training in hypoxia on aerobic and anaerobic capacity [21,22]. On the other hand, few studies have addressed the issue of changes in physical performance before and after a stay at high altitude (>3000 m) [8,23]. Poor athlete health, including low levels of iron and ferritin or red blood cell indices, is associated with decreased physical performance [24], while an increase in red blood cell mass improves aerobic capacity [25,26]. Altitude training, due to the intake of less oxygen during respiration, can be conducted to improve cardiorespiratory indices. The optimal altitude for this type of training is unknown; however, most scientific studies have been performed at intermediate and high altitudes (2000–3000 m) [27], where increases in erythropoietin levels have been observed with minimal side effects [28,29]. Altitudes above 3000 m can result in the loss of training intensity and subsequent muscle atrophy [30]. The main reasons for hypoxia-induced reduction in body and muscle mass are low energy intake, increased energy expenditure, unfavorable climatic conditions and the type of muscle fiber structure [31,32,33]. At altitudes above 5000 m above sea level, 60–70% of weight loss concerns lean body mass [34,35]. A reduction in lean body mass negatively affects aerobic capacity [36], muscle strength [37] and immune function [38], which can increase the risk of disease and injury under these extreme conditions. A decrease in muscle mass, the reduction of the fiber cross-sectional area and mitochondrial density in muscle fibers are the main causes of deterioration in physical performance after chronic hypoxia [8]. HIF-1, in response to hypoxia and increased free radical levels, initiates mechanisms to reduce free radicals in mitochondria [39]. Mechanisms induced by HIF-1 reduce oxidative stress, along with the apoptosis of muscle fibers [39]. However, compensation for the effects of hypoxia by HIF-1 is limited by the dose of hypoxia. Exercise capacity, including aerobic capacity, is impaired under hypoxic conditions, and maximal oxygen consumption ($\dot{V}$O_2max_) decreases by 6% for every 1000 m of elevation (from 300 to 2800 m) [40]. Humans demonstrate a compensatory physiological and biochemical response to staying at high altitudes, especially in its early stages [41]. An increase in the concentration of red blood cells in the blood is an important feature of acclimatization to high altitudes [42,43]. Hypoxic kidney and liver cells release the hormone erythropoietin, which, in turn, stimulates the bone marrow to produce red blood cells [44]. Prolonged exposure to high and very high altitudes (in the range of 2800 to 5800 m) increases hematocrit levels, and this increase is accompanied by an increase in hemoglobin levels and blood viscosity [45]. Proper nutrition, hydration and appropriately selected supplementation (of micronutrients that are deficient in high-mountain conditions, antioxidants and ingredients that improve exercise performance) all help optimize mountain activities and can help prevent nutritional deficiencies that negatively affect health, cognition and physical performance [46,47,48].

The purpose of the study was to determine the effect of a 3-week stay at an altitude above 3000 m on the physical performance and nutritional indices of the health status of mountaineers participating in high-altitude expeditions. The following research hypotheses were put forward: (1) staying in high altitude conditions improved the performance of climbers due to improved red blood cell indices and (2) the high-altitude expedition negatively affected health indices. The results of the study will serve to deepen the knowledge of physiological and biochemical adaptations of high-altitude climbers and the possibility of using these adaptations to increase athletic performance, as well as appropriate training programming.

## 2. Materials and Methods

### 2.1. Study Participants

The study group consisted of 17 Polish male mountaineers between the ages of 23 and 40, who participated in mountain expeditions at least once a year, during which they stayed at altitudes above 3000 m for at least 3 weeks. Individuals who qualified for the study were members of high-mountain clubs affiliated with the Polish Mountaineering Association. The group’s anthropometric and BMI data are shown in Table 2.

The climbers participating in the study were characterized by extensive climbing experience (10 ± 5 years) in sport climbing and mountaineering. The average climbing level of participants, as measured by the IRCRA (The International Rock Climbing Research Association) scale, was 20.69 ± 2.8, which, according to the proposed criteria, places them in the advanced (level 3) category [49]. The climbers who qualified for the study spent an average of 8 ± 3 h per week training. The climbers stayed at an altitude of 3000–8167 m above sea level during their expedition. In total, the high-altitude expeditions lasted an average of 34 ± 6 days, of which participants spent an average of 22 ± 4 days actively climbing in the mountains and 11 ± 8 days resting in base camps or mountain towns. Because some of these sites were located above 3000 m, the participants spent a total of 26 ± 6 days at altitudes above 3000 m.

The mountaineers recruited for the study traveled in small groups or individually to pursue their mountain aims, which are shown in Table 3.

Prior to the start of the project, the mountaineers had a consultation with a sports medicine doctor, during which they were qualified to participate in the project based on the results of previous tests (ECG, blood and urine tests). The presence of chronic diseases and age over 45 were disqualifying factors for participation in the project. The Bioethics Committee of the Cracow Regional Medical Chamber approved the research project (67/KBL/OIL/2021), which was conducted in accordance with the Declaration of Helsinki. After learning about the risks and benefits of taking part in the study, all climbers gave written informed consent to participate.

### 2.2. Study Design

#### 2.2.1. Analysis of Aerobic and Anaerobic Capacity

Aerobic capacity was assessed during a test with gradually increasing load, performed until failure due to extreme fatigue, while anaerobic capacity was assessed during the Wingate test for the lower and upper extremities. Exercise tests were performed twice. The first test was performed about two weeks before the expedition, and the second up to four days after returning from the expedition at an altitude of about 383 m above sea level. The study was conducted as soon as possible after return to catch hypoxia-induced changes in health, body composition and physical performance.

1. Aerobic performance

The test was performed on an h/p/Cosmos Saturn mechanical treadmill (h/p/Cosmos Sports & Medical GmbH, Germany) according to the following scheme: a 2 min recording of baseline cardiorespiratory indices in a standing position, a 4 min run at a constant speed of 8 km/h and a treadmill incline angle of 1°, followed by a 1.1 km/h increase in running speed every 2 min until a running speed of 16.8 km/h was reached. In the remaining part of the test, the running speed did not change, and an increase in exercise intensity was obtained by raising the angle of the treadmill by 1° every 2 min. The test was continued until exhaustion, subjective to the test participant. Respiratory indices were monitored continuously using a Cortex Metalyzer 3B ergospirometer (CORTEX Biophysik GmbH, Leipzig, Germany), and heart rate was recorded using a Polar S-410 cardiac monitor (Polar-Electro, Helsinki, Finland). A number of cardiorespiratory indices were recorded during the test, including heart rate (HR), minute oxygen uptake ($\dot{V}$O_2_), minute carbon dioxide excretion ($\dot{V}$CO_2_), minute pulmonary ventilation ($\dot{V}$E), percentage of oxygen and carbon dioxide in exhaled air (FEO_2_ and FECO_2_) and respiratory quotient (RER). Based on the changes in respiratory indices during the test, the magnitude of maximum minute oxygen uptake ($\dot{V}$O_2max_) and the level of ventilation threshold II were determined. The maximum percentage of carbon dioxide in exhaled air (FECO_2_), the minimum value of the respiratory equivalent for carbon dioxide ($\dot{V}$E/$\dot{V}$CO_2_) and a sharp increase in minute pulmonary ventilation ($\dot{V}$E) were used as criteria for determining the second ventilation threshold.

2. Anaerobic performance

This test was performed in a sitting position without getting up from the saddle on a Monark 894e cycloergometer for the lower limbs (Monark Sports & Medical, Vansbro, Sweden) and Monark 834e for the upper limbs (Monark Sports & Medical, Vansbro, Sweden) in a modified 20-s version. The test participants’ task was to perform crank turns at maximum rhythm throughout the test. Both tests were preceded by warm-ups, with the lower limb test lasting 5 min with a 100-watt load, and the upper limb test lasting 4 min with a 60-watt load. The pedaling rhythm was 60 rpm. During the warm-up for the lower limb test, brief 5 s accelerations of the pedaling rhythm to about 80% of subjective maximum capacity occurred in the 2nd, 4th and 5th min, and in the warm-up for the upper limb test in the 2nd and 4th min, respectively. After the warm-up, the participants performed stretching exercises for 2–3 min, after which the test proper was started. The test load was set at 7.5% and 4.5% of body weight for the lower and upper limb tests, respectively. During both tests, the course of power changes was monitored continuously, which allowed the determination of the following indices: maximum anaerobic power (MAP), total work (W_t_), mean anaerobic power (P_mean_), time to reach maximum power (t_r_) and time to maintain maximum power (t_m_).

#### 2.2.2. Analysis of Health Status

1. Anthropometric measurements and body composition analysis

The body height of the participants was measured before the expedition using a Seca 217 anthropometer (Seca GmbH & Co., KG, Hamburg, Germany) with an accuracy of 1 mm. The climbers’ body weight was determined using an InBody 120 body composition analyzer (Inbody Bldg., Seoul, Republic of Korea) in the morning after a standardized meal between 7:45 and 8:30 a.m.

Body composition analysis was performed using dual-energy X-ray absorptiometry (DXA), with the Lunar iDXA™ instrument (GE HealthCare, Chicago, IL, USA). The DXA method used the phenomenon of attenuation occurring when a beam of ionizing radiation passes through various tissues of the body. The radiation dose was very low, so the test was safe. The Lunar iDXA used fan beam technology with a 64-channel solid-state detector. Each body composition analysis provided information on body mass (BM) [kg] and body mass index (BMI), as well as individual components of body structure: body fat (FM) [% and kg], fat free mass (FFM) [% and kg], lean body mass, i.e., body mass without body fat and bone mineral (LBM) [% and kg], bone mineral content (BMC) [g] and bone mineral density (BMD) [g/cm^2^]. The amount of LBM [kg] in both the upper limbs (arms) and lower limbs (legs) was also assessed. The values obtained additionally allowed for the estimation of the sum of appendicular lean soft tissue (ALST) weight. The ALST index was the sum of the lean body mass of the upper and lower limbs (ALST = LBM arms + LBM legs). With this, muscle mass (MM) [kg and %] was calculated. Kim’s formula was used to calculate muscle mass in kilograms, with a value of 1 for the male gender: MM = (1.13 × ALST) − (0.02 × age) + (0.61 × gender) + 0.97 [50]. Composition analysis and weight measurement were performed twice. The first analysis was performed about two weeks before the expedition, and the second analysis was performed up to four days after returning from the expedition at an altitude of about 383 m above sea level.

2. Blood and urine tests

For health status analysis, blood samples were taken from veins in the elbow pit to determine hematological and biochemical blood indices, i.e., peripheral blood count (hematocrit (HCT), hemoglobin concentration (HGB), mean corpuscular volume (MCV), mean corpuscular hemoglobin (MCH), mean corpuscular hemoglobin concentration (MCHC), red blood cell distribution (RDW), erythrocyte count, platelets count, mean platelet volume (MPV), white blood cell count—leukocytes, leukocyte differentiation: lymphocytes (LYMPH), monocytes (MON), basophils (BASO), eosinophils (EOS), neutrophils (NEU), erythrocyte sedimentation rate (ESR), glucose, total cholesterol (TC), high-density lipoprotein cholesterol (HDL), low-density lipoprotein cholesterol (LDL), triglycerides (TG), creatinine, urea (U), uric acid (UA), total protein, sodium, potassium, chloride, calcium, magnesium, phosphorus, iron, ferritin, vitamin D, vitamin B12, liver enzymes: alanine aminotransferase (ALT), aspartate aminotransferase (AST), acid phosphatase, gamma-glutamyl transpeptidase (GGTP), albumin and total bilirubin. The determinations were carried out in the morning and on an empty stomach, about two weeks before the expedition and within two days after the expedition.

In addition to the blood test, a general urine test was performed, i.e., specific gravity, pH, leukocytes, nitrites, protein, glucose, ketones, urobilinogen, bilirubin, blood (erythrocytes/hemoglobin), color and transparency. The urine for testing was delivered to the laboratory at the same time as the blood draws were performed. Measurements were made by flow cytometry (blood count), kinetic-photometric (ESR), indirect potentiometry (Na, K, chloride), spectrophotometry (total protein, calcium, phosphorus, magnesium, TC, HDL, TG, U, UA, iron, AST, ALT, creatinine, total bilirubin, GGTP, glucose), direct chemiluminescence (vitamin B12, vitamin D, ferritin) and by strip testing (urinalysis). Laboratory determinations were performed using the following devices: Alinity HQ (Abbott, Singapore, Singapore), Roller 20 (Alifax, Polverara, Italy), Alinity C (Abott, Singapore, Singapore), Alinity I (Abbott, Singapore, Singapore) and Atellica 1500 (Siemens, Munich, Germany), and were commissioned as an external service, performed by Alab Laboratories (Warsaw, Poland).

#### 2.2.3. Nutritional Analysis of Diet

The supply of selected macronutrients during the mountaineering expedition was determined by analyzing the whole-day rations obtained using the 3-day food diary method. The expedition participants were asked to note down all the foods, meals and supplements eaten and fluids drunk during 3 days during the expedition (2 days of sports activity, 1 day of rest). These were days of acclimatization and alpine climbing on the way to the summit, and the activities noted during this time were mainly hiking with climbing elements. Climbers were given detailed instructions on how to keep food diaries before leaving for the expedition in order to minimize recording errors. The diaries contained commercially available products, so the necessary data for analysis were read from labels and nutritional tables. In the case of food products such as bars, energy gels and freeze-dried products, climbers were asked to provide the product name, manufacturer and product weight.

The data from the diaries were meticulously entered into the Aliant Dietetic Calculator program (Anmarsoft Marcin Olech, Gdańsk, Poland; version: 85; database: 6.2) for quantitative analysis of the climbers’ diets during the expedition. The program used the “Tables of food composition and nutritional value” [51] as a database, taking into account food recipes and losses due to product processing.

#### 2.2.4. Measurement of Energy Expenditure

Measurements of energy expenditure during the expedition were made by the researchers using a Polar M430 monitor recording heart rate (Polar-Electro, Kempele, Finland), which was recorded 24/7 for 3 days (2 days of sports activity, 1 day of rest). The pulsometers consisted of watches (receiver) and transmitter straps with the Polar H10 sensor (Polar-Electro, Kempele, Finland), worn on the chest at the level of the metasternum in the anterior midline. The watches were placed on the wrist, so that they were adjacent to the body just below the wrist bone. Before the measurement began, the required data were entered into the device for each athlete: gender, age, body weight and height. Before the start of physical activity, the subjects were instructed to put on the transmitter strap, start the physical activity recording option on the device (climbing outdoors), and when the physical activity was over, to turn off the recording by pressing the corresponding button on the watch. In case of recording activity during acclimatization or when reaching the climbing starting point, the training option was not enabled. All climbers were advised not to remove the devices from their wrists for the entire duration of the energy expenditure recording (sleep, daytime activity, physical activity). Energy expenditure was recorded in the Polar Flow app.

#### 2.2.5. Statistical Analysis

Basic statistical measures in the form of arithmetic mean ($\bar{x}$), standard deviation (SD), median (Me) and quartiles (Q1, Q3) were used to characterize the received results. The obtained research results were tested by checking for normal distribution of variables with the W Shapiro–Wilk test. For normal distributions, differences in the mean values of the observed indicators were tested using the Student’s *t*-test for dependent groups, and for other distributions (deviating from the normal distribution), non-parametric statistics were used, with the Wilcoxon paired rank-order test for dependent groups. The significance level was taken as *p* ≤ 0.05. The statistical analysis package Statistica 13.1 (StatSoft, Tulsa, OK, USA) was used for statistical analyses.

A binary classification approach was used to assess the implementation of nutritional standards. For each nutrient, a value of 1 was assigned if the subject’s intake of the nutrient met the recommended standard, and 0 if it did not. Then, based on these data, the percentage of subjects who met the standards for each nutrient was calculated. The analysis was carried out using an Excel spreadsheet (Microsoft Corporation, Washington, DC, USA). The median intake of macronutrients (protein, fat, total carbohydrates), dietary fiber, simple carbohydrates, saturated fatty acids, and micronutrients over 3 days was referred to recommendations for athletes [52,53,54,55,56]. The results were presented in the form of percentages, which made it possible to assess the degree to which the study group implemented the standards.

## 3. Results

The level of indices characterizing the aerobic capacity of climbers (maximum minute oxygen uptake globally and relatively, maximum minute pulmonary ventilation, maximum heart rate, effort duration) decreased after returning from the expedition. The described changes were statistically significant [Table 4].

The indices characterizing the level of the second ventilatory threshold after the expedition did not change significantly, as shown in Table 5. The only statistically significant change was observed in the level of heart rate, which decreased during the study, as measured after the return from the expeditions.

The level of indices characterizing the participants’ anaerobic capacity generally decreased after returning from the expedition [Table 6]. In the lower limb test, maximum anaerobic power and mean anaerobic power expressed in global and relative values decreased. The values of total work and time of maintaining maximum power were also lower during the tests conducted after returning from the expeditions. The time of reaching maximum power decreased, but this change was not statistically significant compared to the other indicators mentioned

In the upper limb test, the changes in the analyzed indices were slightly different. Maximal anaerobic power, both globally and relatively, decreased but the observed changes were not statistically significant. Similarly, changes in the time to reach and maintain maximal power were also not statistically significant. In the upper limb test, the values of mean anaerobic power from the test and total work decreased in a statistically significant way [Table 7].

A prolonged stay in high-altitude conditions resulted in a statistically significant decrease in body weight and muscle mass. There was also a significant decrease in lean body mass in the upper and lower limbs, as well as the ALST index. Analysis of BMI, fat mass, lean mass, muscle mass [%], lean body mass and bone mineral content and bone mineral density showed no significant changes after chronic hypoxia [Table 8].

The analysis of the hematological indices showed a significant increase in the number of leukocytes, erythrocytes and platelets and an increase in the values of hemoglobin and hematocrit, as well as neutrophils and monocytes Table 9. A significant decrease in total cholesterol, HDL cholesterol, total bilirubin, albumin and total protein was observed. There was a statistically significant increase in the levels of total acid phosphatase and inorganic phosphorus. All of the urine indicators remained within the reference values both before and after the expedition.

The median energy demand of the study participants was 3897.95 ± 521 kcal, and the energy supply was 2715.71 ± 794 kcal. The climbers were equipped with supplementation of 325 ± 220 kcal on average, which accounted for 11.99% of their energy supply. Generally, only one person, i.e., 5.88% of the respondents, covered their needs in accordance with the standard. Most climbers (94.14%) met the carbohydrate requirement, and 58.82% met the protein requirement. All climbers met the requirements for fats, and 23.53% of them exceeded the normative values. The median energy and nutritional value of the diet based on analysis of the food diaries is shown in Table 10. Compared to the standards for athletes [53,54,55,56], mountaineers did not provide adequate amounts of potassium, calcium, magnesium, iron, zinc, selenium and iodine, as well as vitamins A, D, C, folate and vitamin K.

Table 11 shows the percentages of climbers whose results of nutritional indices of health status after chronic hypoxia were in accordance with laboratory standards and which were realizing the requirements for selected macro- and micronutrient components of the diet. Most climbers had test results in line with the laboratory standard. The exception is the climbers’ vitamin D levels, which were too low in 52.94% of those tested. Most climbers covered their macronutrient requirements with diet and supplementation. A small percentage of the subjects (<50%) covered the needs for iron, folate, potassium, calcium, vitamin D, vitamin C and vitamin A. If there was an inadequate supply of iron, folate and vitamin D, this was reflected in the blood test results. None of the climbers surveyed provided adequate calcium and vitamin K relative to standards.

## 4. Discussion

### 4.1. Main Findings

In our study, a decrease in aerobic capacity in climbers was observed, expressed as a statistically significant decrease in maximal oxygen uptake ($\dot{V}$O_2max_), maximum minute pulmonary ventilation ($\dot{V}$E_max_), a decrease in the duration of the performance test (t_max_) and a decrease in the value of the maximal heart rate (HR_max_). Similar results were observed by Hoppeler et al. [8], describing a 5% decrease in aerobic capacity expressed as $\dot{V}$O_2max_ in subjects after a minimum of 6 weeks of exposure above 5200 m. A study by Szymczak et al. [23] showed no significant changes in aerobic capacity parameters in alpinists after 4 weeks of being in hypoxia at an average altitude of 4900 m. In our study, there were no significant changes in the indices characterizing the level of second ventilatory threshold. Changes in oxygen uptake at the threshold, minute pulmonary ventilation and time of threshold onset were small and not statistically significant. Different results were obtained in their study by Szymczak et al. [23], who reported a significant decrease in anaerobic threshold and heart rate levels after a month of staying at an average altitude of 4900 m; similarly, in our study, a significant decrease in the value of heart rate at the level of the second ventilation threshold was observed. An interesting observation was made in a study by other authors [40], showing that exercise capacity and aerobic capacity are impaired under conditions of simulated hypoxia, with $\dot{V}$O_2max_ decreasing linearly by 6% for every 1000 m of elevation (from 300 to 2800 m). In our study, $\dot{V}$O_2max_ levels were determined before and after the expeditions, and a 3.95% decrease was noted.

The level of indices characterizing the anaerobic capacity of the subjects generally decreased after returning from the expedition. Such changes are confirmed by studies of other authors [23], who indicate a decrease in total anaerobic work and maximum anaerobic power after 1 month at altitudes above 3500 m (average 4900 m) [23]. Doria et al. [58] observed a decrease in the mechanical and metabolic indices of the Wingate test after a 43-day expedition to the Himalayas (23 days above 5000 m). Grassi and co-workers observed that, after 10 days of acclimatization to 5050 m, muscle power output and blood lactate accumulation were unaffected by chronic hypoxia during short supra-maximal exercise [59,60]. Other studies have shown decreases in lower limb peak power at altitude and after return from high altitude when compared to before departure [61,62]. In our study, there was a statistically significant decrease in MAP (4.91%), average power (5.77%) and total work (5.77%) performed in the test. The change in the time to reach MAP was not statistically significant, while the time to maintain maximum power significantly decreased (6.98%). In the upper limb power tests, the only significant changes were noted in the values of mean anaerobic power and total work, which decreased by 5.73% and 5.74%, respectively. Kayser et al. [63] measured the upper and lower limb maximum alactic anaerobic power of healthy Caucasians both at sea level and during a one month stay at 5050 m. Anaerobic alactic performance was unaltered by a sojourn at very high altitude in this study [63].

In the study, the participants were exposed to adverse environmental conditions while staying at high altitudes (>3000 m) for relatively long periods (>3 weeks). The average climbing speed corresponded to an exercise intensity of 50% $\dot{V}$O_2max_ [64]. This type of effort and the type of mountain activities performed may explain the reduction in muscle strength during the Wingate test [65]. The muscle fibers of the vastus lateralis likely changed from fast-twitch (type 2) to slow-twitch (type 1) fibers [65]. In general, type 1 fibers have lower mechanical power than type 2 fibers, which is the reason behind the reduced ability of leg muscles to perform extremely powerful movements that require mainly anaerobic sources [66]. A reduction in the number of fast-twitch fibers rebuilt at altitude may result in a decrease in anaerobic capacity at sea level, which we observed in climbers returning from high-altitude expeditions. The lower strength of contraction after staying at high altitude would explain the reduced maximal anaerobic power and total anaerobic work observed in the Wingate test in our study. Additionally, the decrease in muscle mass observed in our study may have led to weaker muscles and reduced muscle function [67] and power [61].

In our own study, there was a statistically significant decrease in body mass (1.56%) and muscle mass (1.84%) in a group of alpinists staying a minimum of 3 weeks at altitudes above 3000 m. These results are consistent with a study by Hoppeler et al. [8], in which the researchers noted a 5% decrease in size of body mass in climbers after a minimum 6-week stay at altitudes > 5200 m. A significant reduction in muscle fiber size (−20%) and loss of muscle oxidative capacity (−25%) were observed. Oxygen supply to muscle mitochondria was found to improve after exposure to high altitude. Body mass loss has been considered to be due to both the loss of muscle mass and loss of total body fat [8]. Hypoxia occurring at high altitudes (>5500 m) leads to a loss of lean body mass of up to 15% [68], with consequent muscle weakness and reduced muscle function [67] and power [61]. Also, Bondi et al. [69] showed a reduction in muscle mass in participants trekking in the Himalayas at an altitude of 1070–5143 m over 19 days. A study by Szymczak et al. [23] showed no significant changes in lean body mass in alpinists after 4 weeks of being in hypoxia at an average altitude of 4900 m. The dose of hypoxia experienced by the mountaineers was likely insufficient to cause significant deterioration of their muscle structure, which, in turn, may explain the slight changes in their aerobic indices [23]. In our study, there was a significant decrease in muscle mass (MM), which may have affected the magnitude of maximal minute oxygen uptake. Other data from the literature also showed no significant loss of muscle mass [70,71]. Differences in hypoxic doses may explain this discrepancy. It has been suggested that a minimum hypoxic exposure of 5000 km hours (km·h) [72] is required for the development of hypoxia-induced muscle atrophy [70]. Given the statistically significant loss of muscle mass in climbers, it can be supposed that the hypoxic dose was sufficient to induce the described change. This appears to be largely dependent on the duration of exposure to hypoxia, with the greatest body mass loss observed during the first weeks at altitude [73,74]. The factors responsible for changes in body composition mainly include increased basal metabolism and negative energy balance (i.e., a mismatch between energy intake and energy expenditure, often increased due to intensive hiking or alpine climbing) [75,76]. In addition to increased basal metabolism, at least at altitudes above 5000 m, a negative energy balance may result from decreased energy intake due to reduced appetite [77] or, in part, from impaired gut function [33]. Meta-analysis of data on body composition and body mass changes at different altitudes revealed that diet (21%) and duration of altitude exposure (20%) determined most of the heterogeneity in weight changes between studies [12]. The studies that revealed the largest cumulative average weight reductions at high altitude ranged from 22 to 42 days [12]. In our study, as many as 94.12% of the subjects did not meet the energy supply requirement with diet and supplementation.

Our study showed a statistically non-significant decrease in fat mass (from 14.51% to 13.82%) in climbers after a 3-week stay at altitudes above 3000 m. These data are consistent with the results of Kasprzak et al. [78], who conducted a study with men participating in an expedition in the Alps at an altitude of 3200–3616 m for 16 days. The observed decrease in body fat in the study above (from 16.54% to 16.28%) was not statistically significant. Similar results have also been described in other studies [79,80]. On the other hand, the data obtained in our own study are inconsistent with the results of Szymczak et al. [23], who showed a significant reduction in fat mass from 14% of body weight before the expeditions to 11.5% after 1 month at altitudes above 3500 m (average 4900 m). Other studies have also shown a decrease in body fat after a sojourn at high altitudes [12,34,81,82]. According to the results of a meta-analysis of data on body composition and body mass changes at different altitudes, it is diet and type of exposure (active/passive) that explain 25% and 30% of the heterogeneity in fat mass changes between studies, respectively [12]. The statistically insignificant decrease in body fat observed in our study may have been due to the negative caloric balance observed in climbers.

The analysis of bone mineral content and bone mineral density showed no significant changes after a period of chronic hypoxia in our study. The analysis was based on dual energy X-ray absorptiometry (DXA) measurements. Other studies to assess bone density using the method above were conducted using laboratory animals (mice and rats) [83,84]. Further studies involving humans would need to be performed to compare the results. A study involving mountaineers showed that exposure to a very high altitude (>3700 m) reduced bone mineral density and that the deleterious effects of hypobaric hypoxia on the skeleton were not completely eliminated after 12 months [85].

HIF-induced genetic reprogramming includes, among other things, increased erythropoiesis [86]. In our study, we reported a significant increase in erythrocyte count (4.78%) and an increase in hemoglobin (5%) and hematocrit values (4.98%) in climbers staying above 3000 m for a minimum of 3 weeks. The results obtained are consistent with the study of Reynafarje et al. [87], whose results show a 10% increase in red blood cells after 4 weeks at 4500 m. Similarly, Ferretti et al. [88] noted that staying at altitudes above 5200 m for a minimum of 6 weeks resulted in a significant increase in hemoglobin of 14.1% and hematocrit of 12.2%. Also, the results of Kurdziel et al. show a significant increase in red blood cell, hematocrit and hemoglobin levels in climbers after staying at altitudes above 4900 m for 36 to 44 days [89]. The results of a meta-analysis by Ramussen et al. indicate that, at altitudes above 4000 m, exposure time must exceed 2 weeks to produce a significant effect, and the magnitude of the erythropoietic response depends on the initial volume of red blood cells [90]. In addition, hemoglobin mass returns to baseline values at sea level within 2–3 weeks after descending to sea level [91].

Iron requirements are increased at high altitudes due to enhanced erythropoiesis [92]. Adequate iron stores are necessary to sustain the hypoxia-induced increase in heme synthesis and iron-dependent enzyme production during prolonged exposure at altitude [93]. Our study showed a statistically insignificant decrease in iron and ferritin levels. Kasprzak et al. [78] noted a significant decrease in iron levels in climbers staying at altitudes of 3200–3616 m for 2 weeks. In turn, Kurdziel et al. [89] showed a significant increase in iron and ferritin levels after staying above 4900 m for 36 to 44 days. The researchers also observed significantly reduced hemoglobin content in red blood cells, indicating functional iron deficiency among climbers participating in expeditions to K2 and Broad Peak. This change may be a result of prolonged exposure to a hypoxic environment [89]. The results of our own study indicate a non-significant increase in the hemoglobin content of red blood cells, and these data are inconsistent with the results of the above studies. This may be due to the shorter duration of the expedition and the lower altitudes at which the mountaineers stayed, compared to the results of the Kurdziel et al. study [89]. The iron supply with diet and supplementation in 29.41% of the climbers surveyed in our study was not in accordance with recommendations. The blood iron concentration in 94.12% of climbers after the expedition was normal, while ferritin levels were not adequate in 23.53% of subjects. Due to an insufficient supply of iron and folic acid and a low percentage of people covering their vitamin B12 requirements (58.82%) with diet and supplementation, there is a risk of iron deficiency during or after returning from a high-mountain expedition. Iron deficiency is associated with reduced physical performance in athletes [94], so it is important to monitor iron and ferritin levels after returning from expeditions.

Monocytes, neutrophils and tissue macrophages are responsible for the phagocytosis processes of pathogens [95]. In our study, we observed a statistically significant increase in monocyte (20%) and neutrophil levels (20.67%) after high-altitude expeditions. Similar results were obtained in a study by Pham et al. [96], who showed that the total frequency of monocytes as a percentage of the total number of white blood cells was significantly elevated on the first day of acute exposure at very high altitude (after a night of arrival at 3800 m), compared to values at sea level. However, these studies differed significantly in the time of exposure to altitude. Long-term hypoxia led to adaptive changes in the immune system, including increased numbers of monocytes and neutrophils, which can be interpreted as a response to altitude stress and hypoxia. A recent study showed that exposure to high altitudes with pre-acclimation induces the expression of genes involved in neutrophil activation. This finding indicates that neutrophil activation contributes to an important biological function that promotes human acclimatization at high altitudes [97].

A statistically significant increase in platelet count (17.76%) was observed in our study. These results are consistent with those obtained by Rocke et al. in a study involving 63 healthy lowland volunteers acclimatizing to very high altitude (5200 m) [98] and with those of Hudson et al. who showed a significant and sustained increase in platelet count in 26 healthy individuals 48 h and one week after ascending to very high altitude (3600 m) [99]. Possible explanations for the increase in platelet count that potentially need to be considered include increased physical exertion while at altitude, medication or a change in diet, all of which can interfere with platelet production and function [100]. In contrast, other researchers have observed a decrease in platelet counts after exposure to altitudes of 3200 m and 3771 m [101]. The results of a data meta-analysis by Wang et al. [102] showed that chronic high-altitude hypoxia (≥1 month) results in a significant decrease in platelet count, while acute high-altitude exposure has no significant effect on platelets.

In our study, there was a significant decrease in the serum albumin concentration (0.88%) in climbers after returning from expeditions. These results are consistent with those of Kurdziel et al. [89], who studied climbers participating in expeditions to K2 and Broad Peak. The Smolichev study also confirms the above results [103]. In this study, the relative and absolute content of serum albumin decreased immediately after the ascent and remained low for a month after the descent from 4200 m to 850 m [103]. The body responds to each extreme and abnormal stimulus with a decrease in serum albumin and an increase in serum globulin. The transition from normal atmospheric pressure to conditions of reduced atmospheric pressure and back again is considered a stress, which causes an internal functional reorganization of the entire physiological system of the organism [103]. Apparently, albumin synthesis is impaired under these conditions. Inflammatory conditions, particularly high concentrations of the cytokines interleukin-6 (IL-6) and tumor necrosis factor (TNF-alpha), reduce serum albumin levels [104]. The results of a study conducted by Hartmann et al. [100] indicate that circulating IL-6, IL-1ra and CRP are up-regulated in response to hypobaric hypoxic conditions at high altitude, and moderate increases in these inflammatory markers may reflect significant local inflammation. Our own study showed a statistically significant reduction in total blood protein concentration (5.17%) after 26 ± 6 days above 3000 m, so these results are inconsistent with those of the study described above. The results of the study [103] indicate that, during the first month of the mountaineers’ stay in the Pamir, the protein concentration increased significantly. After four months at an altitude of 4200 m, the concentration of protein in the blood decreased slightly, but remained above the initial level. In the first month after the descent, serum protein concentrations remained high [103]. The levels of acid phosphatase and organic phosphorus, determined in our study, increased significantly, while bilirubin levels decreased (41.15%) after 26 ± 6 days above 3000 m. However, all of the aforementioned indicators remained within reference values both before and after the expedition. The results of the study by Liu et al. [41], on the other hand, indicate an increase in bilirubin levels in subjects subjected to acute exposure from an altitude of 200 m to an altitude of 3648 m (36 h of train travel).

Our study showed a statistically significant reduction in total cholesterol (8.04%) and HDL cholesterol (11.49%), as well as a decrease in LDL cholesterol, but the change was not substantial. Verratti et al. [105] in their study involving 12 climbers who spent 6 days at 900–5895 m observed similar changes, namely, a significant decrease in total cholesterol and LDL levels, while HDL changes were not considerable. Ferezou et al. [106] studied the effects of a high-altitude sojourn on plasma lipids in subjects who spent 3 weeks at base camp below Annapurna IV (4800 m) after 12 days of hiking. The researchers observed a marked and sustained reduction in plasma cholesterol and phospholipids. The low plasma cholesterol levels at high altitude were mainly due to a reduction in low-density lipoprotein (C-LDL) cholesterol [106]. The effects described above likely depend on both altitude hypoxia and physical activity as consequences of hypoxia- and exercise-related adaptations [107]. In our study, the levels of total cholesterol, HDL, LDL fractions and triglycerides in most of the subjects were in accordance with the laboratory standard. This is confirmed by the supply of cholesterol levels with the diet, which was adequate in 82.35% of the subjects. The lipid profile may also have been influenced by the supply of fiber, as well as SFAs, PUFAs and MUFAs. The median fiber supply in the self-reported study was 26.7 g, which is in line with the standards for this nutrient, while the requirement for this nutrient was met by only 58.82% of the subjects. Dietary fiber reduces the absorption of gastric acid molecules, and this is the main mechanism of fiber’s blood cholesterol-lowering effect [108]. The median supply of saturated fatty acids was 11.56%. Current recommendations from The European Food Safety Authority suggest that the supply of SFAs should be as low as possible [54]. In contrast, the position statement of the Academy of Nutrition and Dietetics, Dietitians of Canada and the American College of Sports Medicine [55] suggests limiting SFA supply to <10% of energy requirements. However, the supply of monounsaturated and polyunsaturated fatty acids in the mountaineers’ diets slightly exceeded the supply of saturated fatty acids, which may have affected lipid indices. When replacing 1% of energy from SFAs with PUFAs, MUFAs, complex carbohydrates and/or plant protein, a 4% to 8% reduction in cardiovascular disease (CVD) risk has been reported [109]. The most important thing for reducing LDL cholesterol in the blood is not to reduce dietary cholesterol dietary cholesterol, but to replace SFAs (animal fats) with vegetable fats. Kim et al. [110], in their study, estimated that the risk of death from cancer increased by 4% for every 5% increase in energy from SFAs. Therefore, in the diet of healthy adults, the intake of saturated fatty acids should be limited in cancer prevention [111]. The median supply of simple carbohydrates with the climbers’ diet and supplementation was 16.77% of the diet’s energy value, and in 70.59% of them, the supply was within the norm. All of the subjects had normal fasting glucose results after returning from the expedition.

Our study showed a statistically significant increase in leukocyte levels (18.14%) and a slight increase in vitamin D levels, but these changes were not statistically significant. These data are inconsistent with the results of Kasprzak et al. [78] who observed a significant decrease in 25(OH)D concentrations and a statistically insignificant decrease in leukocyte levels in men after they returned from an expedition to the Alps, during which they stayed at altitudes of 3200–3616 m. In our study, an adequate blood concentration of vitamin D was observed in a small percentage of climbers (52.94%). The supply of this vitamin with the diet was too low (median intake: 2.6 µg), and the percentage of subjects covering the requirement according to standards was only 17.65%. Given how important a role vitamin D plays in the context of the immune system and the fact that its deficiency is associated with a higher risk of anemia [24,112,113], it is important to monitor the concentration of this vitamin before the expedition, adjust the appropriate dose to the current serum concentration and continue supplementation during the expedition [46].

Compared to standards for athletes [50,51,52,53], mountaineers did not provide adequate amounts of potassium, calcium, magnesium, iron, zinc, selenium and iodine, as well as vitamin A, D, C, folate and vitamin K. Moreover, a small percentage of the subjects (<50%) met their needs for iron, folic acid, potassium, calcium, vitamin D, vitamin C and vitamin A, and none of the participating climbers provided adequate amounts of calcium and vitamin K relative to standards. While staying in high-mountain conditions, alpinists should consider including the necessary supplementation of deficient components, especially iron, calcium, potassium, magnesium, zinc, selenium, iodine, B vitamins, as well as vitamin D, C, A, E and K. Appropriate supplementation should be determined individually with specialists—a physician and a nutritionist.

### 4.2. Limitations and Strengths

Our study is one of the few to analyze aerobic and anaerobic capacity, body weight and composition and the hematological and biochemical parameters of climbers’ blood and urine before and after a minimum 3-week stay at altitudes above 3000 m. Our results show how prolonged exposure to hypoxia affects physical performance at sea level. These data can be used by many climbers and alpine tourists planning their expeditions at altitudes above 3000 m.

Limitations of the study include the fact that the climbers’ fitness was tested on average on the third or fourth day after their return from the expedition, as they had to travel from another country and distant mountains to the corresponding laboratory in Poland. This makes it impossible to compare the results with other studies, which differ in the number of days between the end of exposure to hypoxia and when the sea level measurements were taken. The climbers did not constitute a single group, led by a single climbing goal in the territory of a single country and mountain range. Climbers, characterized by the characteristics described in the study, constitute a small but elite group in Poland, hence the several distinct expedition groups, climbing at similar altitudes and with similar sporting goals, but in different parts of the world that qualified for the study. Collecting and encouraging participants was one of the most challenging stages of the ongoing project.

In addition to the level of hypoxia, other factors, such as nutrition and the type of physical activity performed in the mountains, as well as the number of active days versus the number of rest days, may contribute significantly to the observed changes in body composition. Climbing groups active in Peru could have used the downtime between several days of mountain activity to descend to a town at the foot of the mountains to compensate for the negative energy balance there, while expedition groups in Kyrgyzstan, Nepal or Pakistan were dependent on the food they took into the mountains or that the expedition agency offered them. The dose of hypoxia was not measured in our study, but, given the statistically significant loss of muscle mass in climbers, it can be assumed that the hypoxic dose was sufficient to induce the described change. It would be worthwhile to measure this indicator in future studies to be able to clearly assess the value of hypoxia that correlates with the decrease in muscle mass in test subjects.

In the future, it would also be advantageous to improve the conduct of dietary analysis. It would also be worthwhile for a nutritionist to participate in the expedition, so that he or she is in constant contact with the cooks responsible for preparing the meals and has control over the amount of food consumed.

## 5. Conclusions

More than three weeks of hypoxic exposure (>3000 m) had adverse effects on aerobic capacity, lower limb anaerobic capacity and some indices of upper limb anaerobic capacity of the climbers, despite the observed erythropoietic effect. Being in high altitude conditions did not adversely affect the alpinists’ health other than by lowering HDL cholesterol levels. Adverse changes in the body composition of climbers may have worsened physical performance, so in view of the negative environmental factors observed in high altitude conditions and increased metabolism, emphasis should be placed on optimizing energy supply and tailored supplementation to reduce the described effects. To improve the nutritional status of the body both during the expedition and upon return, alpinists should consider necessary supplementation of deficient components. Appropriate supplementation should be determined individually with specialists—a physician and a nutritionist.

## Figures and Tables

**Table 1:** Definitions of altitude and associated physiological changes [14,15].

Altitude	Definition	Physiological Changes
1500–2500 m	Intermediate altitude	Physiological changes detectable. Arterial oxygen saturation > 90%. Altitude illness rare but possible with rapid ascent, exercise and a susceptible individual.
2500–3500 m	High altitude	Altitude illness common during rapid ascents.
3500–5800 m	Very high altitude	Altitude illness common. Arterial oxygen saturation < 90%. Marked hypoxemia during exercise. A total of 5800 m is the highest altitude of permanent human settlements.
>5800 m	Extreme altitude	Marked hypoxemia at rest. Progressive deterioration despite maximal acclimatization. Permanent survival is considered impossible.
>8000 m	“Death zone”	Prolonged acclimatization (>6 weeks) is essential. Most mountaineers require supplementary oxygen to climb safely. Arterial oxygen saturations about 55%. Rapid deterioration is inevitable and time spent above this altitude is strictly limited.

**Table 2:** Anthropometric data and BMI of climbers participating in the research (*n* = 17).

	Men (*n* = 17)	
	$\bar{x}$	SD
Age [years]	30.29	5.8
Body height [cm]	180.47	8.36
Body weight [kg]	74.96	5.03
BMI [kg/m^2^]	22.83	2.1

Abbreviations: $\bar{x}$—mean; SD—standard deviation; BMI—body mass index.

**Table 3:** The mountain goals of mountaineers.

Number of Climbers Participating in the Expedition	Mountain Massif	Country	Mountain Goal
6	Cordillera Blanca	Peru	the 800-m Cruz del Sur route on the La Esfinge rock monolith (5325 m); new route on Ocschapalca (5888 m); Nevado Churup (5495 m); ascents on Artesonraju (6025 m) and Alpamayo (5947 m)
3	Shuijerab, North Karakorum	Pakistan	virgin peaks: Trident Peak (6150 m) and Sakwa Sar (6050 m)
3	Gangotri Valley, Garhwal Himalaya	India	attempted virgin peaks, but failed due to weather conditions (the highest point of the expeditions—5000 m)
2	Himalaya	Western and Northern Nepal	unsuccessful attempt to climb a virgin peak (due to weather); the highest point—5000 m
1	Himalaya	Nepal	Annapurna (8091 m) and Dhaulagiri (8167 m)
1	Himalaya	Nepal	Ama Dablam (6812 m)
1	Lailak Valley, Pamir-Alai	Kyrgyzstan	ascent via Troschenko route on the north face of Ak-su (5217 m)

**Table 4:** Aerobic performance indices measured before and after a stay at high altitude > 3000 m.

Aerobic Performance Indices	Before Chronic Hypoxia		After Chronic Hypoxia		Statistical Test	*p*
	$\bar{x}$	SD	$\bar{x}$	SD		
$\dot{V}$O_2max_ [L/min]	4.07	0.37	3.85	0.39	T	0.0006
$\dot{V}$O_2max_ [mL/kg/min]	54.53	5.96	52.38	5.49	T	0.0232
$\dot{V}$E_max_ [L/min]	149.34	15.88	138.05	14.43	T	0.0003
HR_max_ [beats/min]	190	10	187	11	T	0.0397
t_max_ [min]	20.38	1.95	18.82	2.16	T	0.0001

Abbreviations: $\bar{x}$—mean; SD—standard deviation; T—Student’s *t*-test for variables with parametric distribution; *p*—statistical significance; $\dot{V}$O_2max_—maximum oxygen uptake; $\dot{V}$E_max_—maximum minute pulmonary ventilation; HR_max_—maximum heart rate; t_max_—effort duration.

**Table 5:** Aerobic performance indices associated with the reaching of second ventilatory threshold measured before and after a stay at high altitude > 3000 m.

Aerobic Performance Indices at VT2	Before Chronic Hypoxia		After Chronic Hypoxia		Statistical Test	*p*
	$\bar{x}$	SD	$\bar{x}$	SD		
$\dot{V}$O_2_ at VT2 [L/min]	2.96	0.24	2.9	0.34	T	0.2545
$\dot{V}$O_2_ at VT2 [mL/kg/min]	39.65	3.50	39.45	4.62	T	0.7722
%$\dot{V}$O_2max_ at VT2	73.06	5.65	75.37	4.87	T	0.0642
$\dot{V}$E at VT2 [L/min]	78.35	8.81	80.71	8.46	T	0.2824
HR at VT2 [beats/min]	159	10	155	10	T	0.0358
%HR_max_ at VT2	73.06	5.65	75.36	4.87	T	0.6418
t at VT2 [min]	11.11	2.15	11.08	2.33	T	0.9330

Abbreviations: $\bar{x}$—mean; SD—standard deviation; T—Student’s *t*-test for variables with parametric distribution; *p*—statistical significance; VT2—second ventilatory threshold; $\dot{V}$O_2_ at VT2—maximum oxygen uptake at VT2; %$\dot{V}$O_2max_ at VT2—percentage of maximum oxygen uptake at VT2; $\dot{V}$E at VT2—pulmonary ventilation at VT2; HR at VT2—heart rate at VT2; %HR_max_ at VT2—percentage of maximum heart rate at VT2; t at VT2—effort duration to reach VT2.

**Table 6:** Anaerobic performance indices of the lower limbs measured before and after a stay at high altitude > 3000 m.

Anaerobic Performance Indices (Legs)	Before Chronic Hypoxia		After Chronic Hypoxia		Statistical Test	*p*
	$\bar{x}$/Me	SD/(Q1; Q3)	$\bar{x}$/Me	SD/(Q1; Q3)		
MAP [W]	807.46	73.66	767.83	78.52	T	0.0015
MAP [W/kg]	10.73	0.77	10.4	0.71	T	0.0130
P_mean_ [W]	668.24	49.57	629.66	45.85	T	0.0004
P_mean_ [W/kg]	8.89	0.64	8.54	0.49	T	0.0022
W_t_ [kJ]	13.36	0.99	12.59	0.91	T	0.0004
t_r_ [s]	5.67	0.63	5.46	1.06	T	0.3053
t_m_ [s]	3.69	(2.90; 4.42)	3.44	(2.51; 3.62)	W	0.0024

Abbreviations: $\bar{x}$—mean; SD—standard deviation; Me—median; Q1—first quartile; Q3—third quartile; T—Student’s *t*-test for variables with parametric distribution; W—Wilcoxon rank test for variables with nonparametric distribution; *p*—statistical significance; MAP—maximum anaerobic power; P_mean_—mean anaerobic power; W_t_—total work performed; t_r_—time of reaching maximum power; t_m_—time of maintaining maximum power.

**Table 7:** Anaerobic performance indices of the upper limbs measured before and after a stay at high altitude > 3000 m.

Anaerobic Performance Indices (Arms)	Before Chronic Hypoxia		After Chronic Hypoxia		Statistical Test	*p*
	$\bar{x}$/Me	SD/(Q1; Q3)	$\bar{x}$/Me	SD/(Q1; Q3)		
MAP [W]	556.64	79.45	536.67	90.53	T	0.1346
MAP [W/kg]	7.38	0.8	7.25	0.86	T	0.4472
P_mean_ [W]	439.51	44.65	414.32	47.45	T	0.0015
P_mean_ [W/kg]	6.02	(5.55; 6.15)	5.64	(5.41; 5.81)	W	0.0245
W_t_ [kJ]	8.79	0.89	8.29	0.95	T	0.0015
t_r_ [s]	5.56	(3.99; 8.46)	4.07	(3.46; 7.20)	W	0.0683
t_m_ [s]	3.95	(2.23; 5.02)	2.53	(2.16; 4.55)	W	0.1239

Abbreviations: $\bar{x}$—mean; SD—standard deviation; Me—median; Q1—first quartile; Q3—third quartile; T—Student’s *t*-test for variables with parametric distribution; W—Wilcoxon rank test for variables with nonparametric distribution; *p*—statistical significance; MAP—maximum anaerobic power; P_mean_—mean anaerobic power; W_t_—total work performed; t_r_—time of reaching maximum power; t_m_—time of maintaining maximum power.

**Table 8:** Anthropometric indices measured before and after a stay at high altitude > 3000 m.

Anthropometric Indices	Before Chronic Hypoxia		After Chronic Hypoxia		Statistical Test	*p*
	$\bar{x}$/Me	SD/(Q1; Q3)	$\bar{x}$/Me	SD/(Q1; Q3)		
BM [kg]	74.96	5.03	73.79	5.53	T	0.0149
BMI [kg/m^2^]	22.83	2.10	22.43	1.43	T	0.1606
FM [kg]	9.64	(8.42; 12.62)	9.30	(7.99; 10.47)	W	0.1024
FM [%]	12.80	(11.50; 17.60)	12.90	(11.60; 16.20)	W	0.1626
FFM [kg]	64.51	5.09	63.99	4.90	T	0.1330
FFM [%]	87.78	(83.05; 88.89)	87.66	(84.46; 88.99)	W	0.1488
MM [kg]	33.72	2.91	33.10	2.73	T	0.0071
MM [%]	44.97	2.05	44.88	2.15	T	0.7851
LBM [kg]	61.35	4.83	61.01	4.74	T	0.4437
LBM [%]	83.39	(79.00; 84.64)	83.43	(80.73; 84.69)	W	0.1626
LBM Arms [kg]	8.25	0.76	8.08	0.73	T	0.0224
LBM Legs [kg]	20.72	2.00	20.35	1.80	T	0.0391
ALST [kg]	28.98	2.58	28.43	2.43	T	0.0071
BMC [g]	3.16	0.33	3.16	0.33	T	0.7562
BMD [g/cm^2^]	1.30	0.07	1.29	0.05	T	0.1054

Abbreviations: $\bar{x}$—mean; SD—standard deviation; Me—median; Q1—first quartile; Q3—third quartile; T—Student’s *t*-test for variables with parametric distribution; W—Wilcoxon rank test for variables with nonparametric distribution; *p*—statistical significance; BM—body mass; BMI—body mass index; FM—fat mass; FFM—fat–free mass; MM—muscle mass; LBM—lean body mass; ALST—appendicular lean soft tissue; BMC—bone mineral content; BMD—bone mineral density.

**Table 9:** Health status indices measured before and after a stay at high altitude > 3000 m.

Health Status Indices	Before Chronic Hypoxia		After Chronic Hypoxia		Statistical Test	*p*
	$\bar{x}$/Me	SD/(Q1; Q3)	$\bar{x}$/Me	SD/(Q1; Q3)		
Blood counts						
Leukocytes [10^9^/L]	5.65	1.05	6.68	2	T	0.0066
Erythrocytes [10^12^/L]	5.18	0.31	5.43	0.36	T	0.0007
HGB [g/dL]	15.39	0.86	16.16	0.88	T	0.0004
HCT [%]	47.26	2.72	49.61	3.41	T	0.0025
MCV [fL]	91.34	2.84	91.53	2.8	T	0.7862
MCH [pg]	29.79	1.43	29.87	1.41	T	0.7134
MCHC [g/dL]	32.61	1.08	32.64	1.28	T	0.9402
RDW [%]	13.25	1.00	13.25	0.90	T	0.9986
Platelets [10^9^/L]	203.8	(193.2; 256.8)	240	(222; 260)	W	0.0075
MPV [fL]	8.6	1.56	8.51	1.37	T	0.8161
NEU [%]	55.10	(46.10; 57.27)	57.16	(49.58; 61.15)	W	0.7583
NEU [10^9^/L]	3	(2.75; 3.19)	3.62	(2.5; 5.5)	W	0.0442
LYMPH [%]	34.45	6.51	29.55	10.06	T	0.1687
LYMPH [10^9^/L]	1.81	(1.61; 2.00)	2	(1.83; 2.15)	W	0.0929
MON [%]	8.18	1.79	8.13	2.92	T	0.9267
MON [10^9^/L]	0.45	(0.40; 0.51)	0.54	(0.49; 0.71)	W	0.0064
EOS [%]	3.11	2.12	2.31	1.44	T	0.1393
EOS [10^9^/L]	0.11	(0.08; 0.27)	0.15	(0.10; 0.24)	W	0.9382
BASO [%]	1.11	0.34	0.84	0.58	T	0.1018
BASO [10^9^/L]	0.06	(0.05; 0.07)	0.06	(0.04; 0.08)	W	0.6592
ESR [mm/h]	2	(2; 2)	2	(2; 5)	W	0.0759
Electrolytes						
Sodium [mmol/L]	140.41	1.09	140.71	2.02	T	0.6071
Potassium [mmol/L]	4.33	0.16	4.42	0.24	T	0.1139
Chlorides [mmol/L]	102	(100; 104)	104	(102; 104)	W	0.2115
Calcium [mmol/L]	2.36	0.12	2.36	0.13	T	0.9379
Magnesium [mmol/L]	0.85	0.04	0.85	0.08	T	0.8599
Lipid profile						
TC [mmol/L]	4.49	0.79	4.13	0.77	T	0.0197
LDL [mmol/L]	2.59	0.66	2.37	0.6	T	0.0942
HDL [mmol/L]	1.56	0.25	1.38	0.3	T	0.0162
TG [mmol/L]	0.70	(0.57; 0.81)	0.62	(0.55; 0.99)	W	0.2659
Kidney parameters						
Creatinine [μmol/L]	78.83	9.8	75.20	10.48	T	0.1855
Urea [mmol/L]	5.23	0.7	4.85	1.2	T	0.3155
Uric acid [μmol/L]	308.17	57.15	327.12	62.90	T	0.1317
Urine pH	5.88	0.47	6.26	0.79	T	0.072
Urine specific gravity	1.02	(1.02; 1.03)	1.02	(1.01; 1.02)	W	0.125
Liver parameters						
AST [U/L]	23	4.42	20.06	4.49	T	0.0710
ALT [U/L]	21	(16; 23)	21	(16; 26)	W	0.3636
Total bilirubin [μmol/L]	19.81	7.98	11.66	5.41	T	0.0001
GGTP [U/L]	17	(16; 19)	19	(17; 20)	W	0.0501
Albumin [g/L]	47	(45;4; 49.3)	45.9	(43.5; 46.6)	W	0.0171
Total acid phosphatase [U/L]	3.6	(3; 4)	4.3	(4.0; 4.4)	W	0.0151
Another indices						
Total protein [g/L]	72.36	3.52	68.62	5.74	T	0.0029
Glucose [mmol/L]	5.01	(4.68; 5.24)	4.88	(4.72; 5.04)	W	0.2659
Inorganic phosphorus [mmol/L]	1.09	0.14	1.26	0.12	T	0.0003
Iron [μmol/L]	26.67	9.01	21.02	7.85	T	0.0567
Ferritin [ng/mL]	70.82	32.19	66.82	36.29	T	0.4492
Vitamin D (25(OH)D) total [ng/mL]	31.76	7.93	31.92	7.31	T	0.8941
Vitamin B12 [pg/mL]	402	(329; 449)	409	(337; 433)	W	0.4074

Abbreviations: $\bar{x}$—mean; SD—standard deviation; Me—median; Q1—first quartile; Q3—third quartile; T—Student’s *t*-test for variables with parametric distribution; W—Wilcoxon rank test for variables with nonparametric distribution; *p*—statistical significance; HGB—hemoglobin; HCT—hematocrit; MCV—mean corpuscular volume; MCH—mean corpuscular hemoglobin; MCHC—mean corpuscular hemoglobin concentration; RDW—red blood cell distribution; MPV—mean platelet volume; NEU—neutrophils; LYMPH—lymphocytes; MON—monocytes; EOS—eosinophils; BASO—basophils; ESR—erythrocyte sedimentation rate; TC—total cholesterol; LDL—low-density lipoprotein cholesterol; HDL—high-density lipoprotein cholesterol; TG—triglycerides; AST—aspartate aminotransferase; ALT—alanine aminotransferase; GGTP—gamma-glutamyl transpeptidase.

**Table 10:** Energy and nutritional value of the daily food ration of mountain climbers in relation to the recommendations for athletes [53,54,55,56].

	Diet of Climbers During the Expedition	Nutritional Recommendations for Athletes
	Me (Q1; Q3)	RDA/AI
	Energy and macronutrients intake	
Energy [kcal]	2700.8 (2023.6; 3036.9)	Daily energy expenditure
Protein [%]	13.8 (11.7; 15.5)	15–20 ^1^
Protein [g]	90.2 (72.4; 105.4)	-
Plant protein [g]	48.90 (36.9; 51.6)	-
Animal protein [g]	33.3 (32.3; 54.4)	-
Protein [g/kg bw]	1.16 (1.01; 1.34)	1.2–2.2 ^1^
Carbohydrates [%]	52.2 (49.8; 54.4)	45–65 ^2^
Carbohydrates [g]	332.2 (268.2; 443.4)	-
Carbohydrates [g/kg bw]	4.37 (3.81; 5.91)	6–12 ^3^
Simple carbohydrates [g]	102 (84.8; 136.3)	-
Simple carbohydrates [%]	16.77 (13.26; 23.33)	<20 ^4^
Fiber [g]	26.7 (21.8; 33.2)	>25 ^4^
Fats [%]	32.8 (27.9; 36)	20–35 ^2,3,4^
Fats [g]	96.6 (78.6; 114.8)	-
Fats [g/kg bw]	1.23 (1.04; 1.56)	0.5–1.5 ^1^
SFA [%]	11.56 (8.42; 14.4)	<10 ^3^; ALAP ^4^
SFA [g]	34.7 (28.3; 38.9)	ALAP ^4^
MUFA [g]	23.2 (10.1; 32.6)	-
PUFA [g]	12.4 (10; 12.8)	ALA 0.5%, LA 4%
Cholesterol [mg]	225.2 (144; 271.5)	<300 ^5^
	Micronutrients intake	
Sodium [mg] (AI)	2832 (2070; 2897.3)	1500–>10,000 ^2^
Potassium [mg] (AI)	2039.7 (1358.1; 2715.4)	3500 ^4^–4700 ^2^
Calcium [mg]	445.3 (349.4; 611)	1000 ^1^–2000 ^3^
Phosphorus [mg]	866.2 (587.2; 901.9)	700 ^1^–1500 ^2^
Magnesium [mg]	278.8 (201.5; 398.7)	420 ^1^
Iron [mg]	7.8 (4.8; 10.4)	11 ^4^
Zinc [mg]	5.4 (4.8; 7.1)	11–15 ^2^
Copper [mg]	1 (0.7; 1.7)	0.9 ^2^–1.6 ^4^
Selenium [µg]	5.1 (1.6; 12.2)	50–55 ^2^
Iodine [µg]	22.8 (13.8; 39.1)	120–150 ^2^
Vit. A [µg]	729.2 (473.1; 1570.8)	900 ^1^
Vit. D [µg]	2.6 (1.2; 4.8)	15 ^2,4^
Vit. E [mg]	14.2 (9.9; 17.8)	15 ^1,2^
Vit. C [mg]	90.5 (74.5; 164.5)	110 ^4^–200 ^2^
Vit. B_1_ [mg]	1.4 (1.2; 1.7)	1.2 ^1^
Vit. B_2_ [mg]	1.4 (0.8; 2.2)	1.3 ^1,4^
Vit. B_3_ [mg]	13.7 (5.1; 20.1)	14–20 ^2^
Vit. B_6_ [mg]	1.8 (1.2; 2.3)	1.5–2 ^2^
Vit. B9 [µg]	325.4 (203.9; 477.1)	400 ^1,2^
Vit. B_12_ [µg]	2.8 (1.6; 4.1)	2.4 ^1^–4 ^4^
Vit. K [µg]	4.2 (2.2; 14.4)	120 ^1^–700 ^2^

Abbreviations: ^1^—[56]; ^2^—[53]; ^3^—[55]; ^4^—[54]; ^5^—[57]; Me—median; Q1—first quartile; Q3—third quartile; RDA—recommended dietary allowance; AI—adequate intake; ALA—α-linolenic acid; ALAP—as low as possible; bw—body weight; LA—linoleic acid; MUFA—monounsaturated fatty acid; PUFA—polyunsaturated fatty acid; SFA—saturated fatty acids.

**Table 11:** Percentage of climbers whose selected test results of nutritional indices of health status after chronic hypoxia were in accordance with laboratory standards and percentage of climbers realizing the requirements for selected macro- and micronutrient components of the diet.

Nutritional Indices of Health Status After Chronic Hypoxia	Percentage of Climbers Whose Test Results of Health Indicators Were Consistent with Laboratory Standards [%]	Macro- and Micronutrients of the Diet	Percentage of Climbers Realizing the Requirement for a Given Dietary Nutrient [%]
Erythrocytes [10^12^/L] HGB [g/dL] HCT [%] MCV [fL] MCH [pg] MCHC [g/dL] Iron [μmol/L] Ferritin [ng/mL] Vitamin B12 [pg/mL]	100 100 76.47 94.12 94.12 70.59 94.12 76.47 94.12	Iron [mg] Vitamin B9 [µg] Vitamin B12 [µg]	29.41 35.29 58.82
Sodium [mmol/L]	100	Sodium [mg]	100
Potassium [mmol/L]	100	Potassium [mg]	23.53
Calcium [mmol/L]	82.35	Calcium [mg]	0
Magnesium [mmol/L]	100	Magnesium [mg]	11.76
Vitamin D (25(OH)D) total [ng/mL]	52.94	Vitamin D [µg]	17.65
TC [mmol/L]	82.35	Cholesterol [mg]	82.35
LDL [mmol/L]	82.35	Fibre [g]	58.82
HDL [mmol/L]	88.24	Simple carbohydrates [%]	70.59
TG [mmol/L]	88.24	Vitamin C [mg]	35.29
Glucose [mmol/L]	100	Vitamin E [mg]	52.94
		Vitamin A [µg]	41.18

Abbreviations: HGB—hemoglobin; HCT—hematocrit; MCV—mean corpuscular volume; MCH—mean corpuscular hemoglobin; MCHC—mean corpuscular hemoglobin concentration; TC—total cholesterol; LDL—low-density lipoprotein cholesterol; HDL—high-density lipoprotein cholesterol; TG—triglycerides.

## Data Availability

Data are contained within the article.

## Abbreviations

The following abbreviations are used in this manuscript:

ALST	appendicular lean soft tissue
ALT	alanine aminotransferase
AST	aspartate aminotransferase
BASO	basophils
BM	body mass
BMC	bone mineral content
BMD	bone mineral density
BMI	body mass index
bw	body weight
EOS	eosinophils
ESR	erythrocyte sedimentation rate
FFM	fat free mass
FM	fat mass
GGTP	gamma-glutamyl transpeptidase
HCT	hematocrit
HDL	high-density lipoprotein cholesterol
HGB	hemoglobin
HR	heart rate
HR_max_	maximum heart rate
LBM	lean body mass
LDL	low-density lipoprotein cholesterol
LYMPH	lymphocytes
MAP	maximum anaerobic power
MCH	mean corpuscular hemoglobin
MCHC	mean corpuscular hemoglobin concentration
MCV	mean corpuscular volume
MM	muscle mass
MON	monocytes
MPV	mean platelet volume
NEU	neutrophils
p	statistical significance
P_mean_	mean anaerobic power
RDW	red blood cell distribution
SD	standard deviation
T	Student’s *t*-test for variables with parametric distribution
TC	total cholesterol
t_m_	time of maintaining maximum power
t_max_	effort duration
tr	time of reaching maximum power
$\dot{V}$E	pulmonary ventilation
$\dot{V}$E_max_	maximum minute pulmonary ventilation
$\dot{V}$O_2max_	maximum oxygen uptake
VT2	second ventilatory threshold
W	Wilcoxon rank test for variables with nonparametric distribution
W_t_	total work performed
$\bar{x}$	mean
